# Study protocol for a cluster-randomized trial to compare human papillomavirus based cervical cancer screening in community-health campaigns versus health facilities in western Kenya

**DOI:** 10.1186/s12885-017-3818-z

**Published:** 2017-12-06

**Authors:** Megan J. Huchko, James G. Kahn, Jennifer S. Smith, Robert A. Hiatt, Craig R. Cohen, Elizabeth Bukusi

**Affiliations:** 1 Duke University, Global Health Institute and Department of Obstetrics and Gynecology, 310 Trent Drive, Room 204, Durham, NC 27708 USA; 2 Department of Epidemiology and Biostatistics, University of California San Francisco, Box 0560, San Francisco, CA 94143-0560 USA; 3 Department of Epidemiology, University of North Carolina, 2103 McGavran-Greenberg Hall Campus, Box# 7435, Chapel Hill, NC 27599-7435 USA; 4 Department of Obstetrics, Gynecology and Reproductive Sciences, University of California, Box 1280, 560 Mission Street, 3rd Floor, San Francisco, CA 94143 USA; 5 Kenya Medical Research Institute, Center for Microbiology Research, P.O. Box 54840 00200, Mbagathi Road, Nairobi, Kenya; 6 Department of Obstetrics and Gynecology, University of Nairobi, P.O. Box 54840 00200, Mbagathi Road, Nairobi, Kenya

**Keywords:** Cervical cancer screening, Community health campaigns, Kenya, HPV self-collection, Implementation science

## Abstract

**Background:**

Despite guidelines for cervical cancer prevention in low-resource countries, a very small proportion of women in these settings undergo screening, and even fewer women are successfully treated. Using pilot data from western Kenya and World Health Organization recommendations, we developed a protocol to implement evidence-based cervical cancer screening and linkage to treatment strategies to the rural communities. We describe the protocol for a cluster-randomized trial to compare two implementation strategies for human-papillomavirus (HPV)-based cervical cancer screening program using metrics described in the RE-AIM (reach, efficacy, adaption, implementation and maintenance) framework.

**Methods:**

The study is a three-year, two-phase cluster-randomized trial in 18 communities in western Kenya. During Phase 1, six control communities were offered screening in health facilities; and six intervention communities were offered screening in community health campaigns. Screening was done with human-papillomavirus testing through self-collected specimens. Phase 1 ended and we are working in partnership with communities to further contextualize the implementation strategy for screening, and develop an enhanced linkage to treatment plan. This plan will be tested in an additional six communities in Phase 2 (enhanced intervention). We will compare the reach, efficacy, cost-effectiveness and adaptability of the implementation strategies.

**Discussion:**

Effective low-cost cervical cancer prevention technologies are becoming more widely available in low- and middle-income countries. Despite increasing government support for cervical cancer prevention, there remains a sizeable gap in service availability. We will use implementation science to identify the most effective strategies to fill this gap through development of context-specific evidence-based solutions. This protocol design and results can help guide implementation of cervical cancer screening in similar settings, where women are most underserved and at highest risk for disease.

**Trial registration:**

This trial is registered at ClinicalTrials.gov, NCT02124252.

**Electronic supplementary material:**

The online version of this article (10.1186/s12885-017-3818-z) contains supplementary material, which is available to authorized users.

## Background

Despite the fact that cervical cancer is highly preventable through vaccination and organized screening programs, over 500,000 women worldwide are diagnosed with the disease every year [1]. About 9 out of 10 cervical cancer deaths occur in low-resource countries, with a particularly high burden in sub-Saharan Africa, where the mortality rate is 85% [2, 3]. The inequality between high and low-resource countries is mainly due to lack of screening in low-resource countries, which lack the health care infrastructure required for the cytology-based screening programs that have dramatically reduced the disease burden in wealthier countries. The World Health Organization (WHO) recommends alternative cervical cancer prevention techniques and protocols for low-resource countries that employ low-cost or simple-to-use screening technologies [4]. One such strategy – high-risk human papillomavirus (HPV) testing– has been shown to reduce the incidence and mortality from cervical cancer when coupled with outpatient treatment for women with HPV-positive results [5].

In addition to effective screening tools, the impact of cervical cancer prevention programs depends on two main context-specific factors: (1) women’s access to screening and (2) successful acquisition of treatment for women who screen positive. Access to both screening and treatment is most challenging in poor rural areas, due to geographic and infrastructure constraints [6, 7]. Most health care in rural areas takes place in small health facilities with limited space, staffing, and equipment, making it challenging to implement same-day “screen & treat” strategies that have been proposed to overcome barriers to treatment access [8]. So, despite the development of guidelines for cervical cancer screening that employed evidence-based technologies and lower resource protocols, the lack of rigorously tested, context-specific implementation strategies has left a gap between policy and practice.

In order to develop a context-specific, sustainable implementation strategy, we undertook formative work to identify local barriers and facilitators for cervical cancer screening in government-supported health facilities in rural western Kenya, an area of East Africa with a high cervical cancer burden and screening rates as low as 3% [9, 10]. We found that access to screening was limited by lapses in service availability and lack of clinic attendance for preventive care [11]. When services were available, both providers and patients found the need for a pelvic exam limited the acceptability of cervical cancer screening. Based on the facility-based barriers, we developed and piloted a highly successful community health campaign model for screening, consisting of outreach followed by a brief campaign held in a central site in the community, offering on-site screening and referrals for treatment [12]. An advantage of community-based screening is that only screen-positive women need to visit health facilities for follow-up care, reducing the visit burden for both woman and facilities, and allowing resources to be directed toward strategies to increase treatment uptake, such as intensified follow-up, transportation assistance or mobile units that bring treatment to remote villages. Thus, as has been seen in other health services [13–15], by combining community-screening with enhanced linkage strategies, our approach could maximize the health impact by increasing the number of women screening *and* the proportion successfully accessing treatment.

To address the reluctance around pelvic exams, we chose to offer screening with self-collected specimens for HPV testing, an evidence-based strategy that would eliminate pelvic exams for initial screening, further increasing screening acceptance and efficiency. We developed a study protocol that will allow us to compare two context-specific implementation strategies for an HPV-based cervical cancer prevention program through a cluster-randomized trial of HPV-based cervical cancer screening in community-health campaigns versus health facilities using the RE-AIM framework [16, 17]. This paper describes the study protocol (V 3.0, 20 July 2017) and the plan to evaluate the adaptability, comparative effectiveness and cost-effectiveness of these two strategies.

## Methods

### Study design and setting

The study is a two-phase cluster randomized trial in western Kenya to evaluate reach, effectiveness, cost-effectiveness and maintenance of two implementation strategies for a cervical cancer prevention protocol that consists of four critical, evidence-based components:

1. **HPV and cervical cancer outreach and education** *.* In western Kenya, we found that women’s baseline knowledge and perception of cervical cancer risk is low; a brief educational intervention provided by community health workers in primary care clinics improved these baseline factors and increased women’s intention to screen [18].
2. **HPV-testing using self-collected specimens with referral for treatment based on a single positive result.** An HPV-based screening strategy is effective at reducing incidence of cervical pre-cancer and invasive cancer when women with screen-positive results undergo cryotherapy [5, 19, 20]. Self-collected specimens are highly accurate, with comparable results to provider-collection for the detection of high-grade cervical precancer [21–23] Women have consistently found self-sampling acceptable and preferable to provider-testing [24–26]; this finding has been supported in studies from sub-Saharan Africa [27, 28]. Studies in various countries have shown that a self-collection strategy increases screening uptake by women not attending clinics [29–34].
3. **Notification of screening results using text messaging.** Based on our prior experience with mobile health interventions [35, 36] and the high rates of cell phone use in western Kenya [37], HPV test results were sent to all women via text message with instructions about appropriate follow-up as recommended by the Kenya Ministry of Health guidelines.
4. **Treatment with cryotherapy unless contraindicated by cervical exam.** Cryotherapy is a low-cost, effective treatment method that can be safely carried out by mid-level providers in low-resource settings [38, 39]. Women who are not candidates for cryotherapy (i.e. lesions too large or abnormal cervical anatomy) were offered Loop Electrosurgical Excision Procedure carried out in the County Hospital. Together, HPV testing followed by cryotherapy for women who test positive reflect the current WHO recommendations [40].

In Phase 1 of this study, we compared two implementation strategies that incorporated these four evidence-based elements of screening. Based on our preliminary data, we found that reaching and attending a health facility for preventive care was a significant barrier to screening for many women. Therefore, the main objective was to compare a model offering screening in brief, high throughput community health campaigns to that of a standard of care in which screening was offered in local health facilities using the metrics defined in the modified RE-AIM framework.

**Fig. 1:**
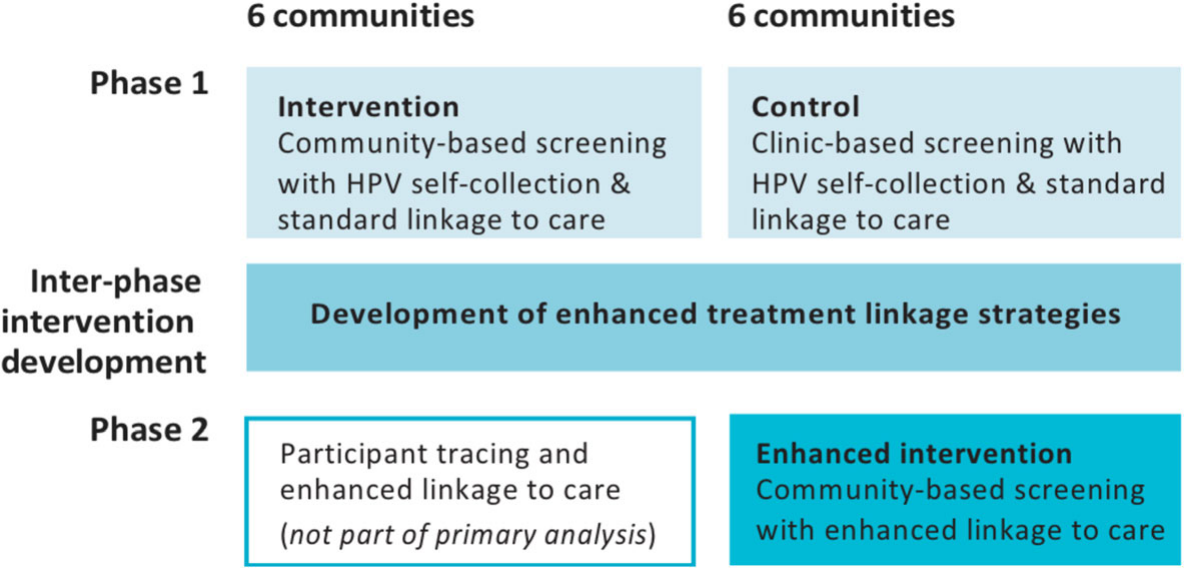
Two-phase cluster-randomized trial design

In Fig. 1, we present an overall schema of the cluster-randomized trial. In Phase 1, six communities were randomized to the intervention: HPV screening carried out in **community health campaigns**. The remaining six were comparison communities: HPV testing offered in **government health facilities**. HPV-test positive women in all communities were referred to the County hospital for immediate treatment, which is considered standard linkage to treatment.

Development of an enhanced strategy for linkage to treatment: After Phase 1, we have an “inter-phase intervention development” period in which we are evaluating the results from the trial. The outcomes from the qualitative and quantitative measures will be used to refine the screening intervention using context-specific details and develop an enhanced strategy for linkage to treatment. Although we have identified factors that enable and inhibit women’s access to treatment, we chose to wait until after Phase 1 for the development of the enhanced strategy for linkage to treatment in order to truly work in partnership with the community. The delayed development of the linkage to treatment strategy has allowed us obtain a baseline measure of the efficacy of standard referrals and identify factors that would influence women’s access to care in this setting.

After developing the strategy for enhanced linkage, we will pilot and then test the linkage to treatment strategy as part of an “enhanced intervention” in the six communities that served as controls in Phase 1. Using these communities for the enhanced intervention increases the efficiency of the study in two ways: i. we will have done community enumeration and engagement, and ii. we will compare linkage to treatment outcomes from these “enhanced intervention” communities to the intervention communities from Phase 1.

### Study activities

#### Study preparation

- **Community enumeration and randomization:** Prior to the initiation of the cluster randomized trial, we characterized the study communities using a combination of census data, health facility information, mapping and prospective demographic data. We identified communities of approximately 7500 people in three sub-counties of Migori County in western Kenya (population: 350,000, 65 government health facilities). Population estimates were calculated using the 2009 Kenyan census data with population growth estimates for 2015 and 2014, a method that was validated through door-to-door enumeration for a recent large-scale community randomized trial in rural western Kenya [41]. Eligible communities had at least one government health facility with capacity to provide HPV testing, support from community leaders for the community outreach and/or health campaigns, accessibility to health centers via a maintained transportation route and sufficient distance from other potential study sites to limit contamination between arms (buffer zones). As our target group is women in rural communities, we excluded urban settings or communities in which the nearest health center is Migori County Hospital and those that were taking part in a cluster-randomized trial of large-scale community-health campaigns for HIV-testing [41]. We conducted unmatched randomization using a random number generator on Stata 10. The unmatched design will allow us to consider the relationship between community-level factors and our outcomes of interest. After communities were chosen, population estimates were refined through household enumeration done by community health workers assigned to villages and sub-locations within the communities.
- **Provider and key stakeholder focus groups:** Clinicians and community health workers (“providers”) within the community health campaigns and health facilities participated in focus group discussions to provide baseline data about perceived barriers and strategies to facilitate HPV screening uptake during the planning and implementation adaptation period of the study. Although we sought key stakeholder input throughout the development of the implementation strategies, we held three focus group discussions with key stakeholders in the intervention and control communities for Phase 1 (community chiefs, leaders of women’s groups, reproductive health coordinator, medical superintendent and Charge Nurse of Migori District Hospital). The goals of these focus groups were to obtain a group perspective on the intervention as planned for their communities, any anticipated challenges and strategies to optimize the screening strategy in both arms. Focus group discussions were analyzed using the theoretical domains framework, which mapped behaviors to intervention strategies, using evidence-based principles of behavior change [42].
- **Training and finalization of the screening protocol.** We used educational modules piloted in western Kenya to provide standardized training in cervical cancer counseling and HPV-self testing to community health workers and clinicians [43]. The community health workers received training in community outreach messaging, delivery of the educational module in the community health campaign setting, and teaching women how to perform self-collection of HPV specimens. In addition to the general training, clinicians had undergone Ministry of Health-supported training to learn the cervical cancer screening protocol, including follow-up and pre-treatment exams. Two nurses who had undergone cryotherapy training were identified and supervised for ten cryotherapies at the County Hospital prior to study initiation.

#### Cluster randomized trial: Phase 1

**Fig. 2:**
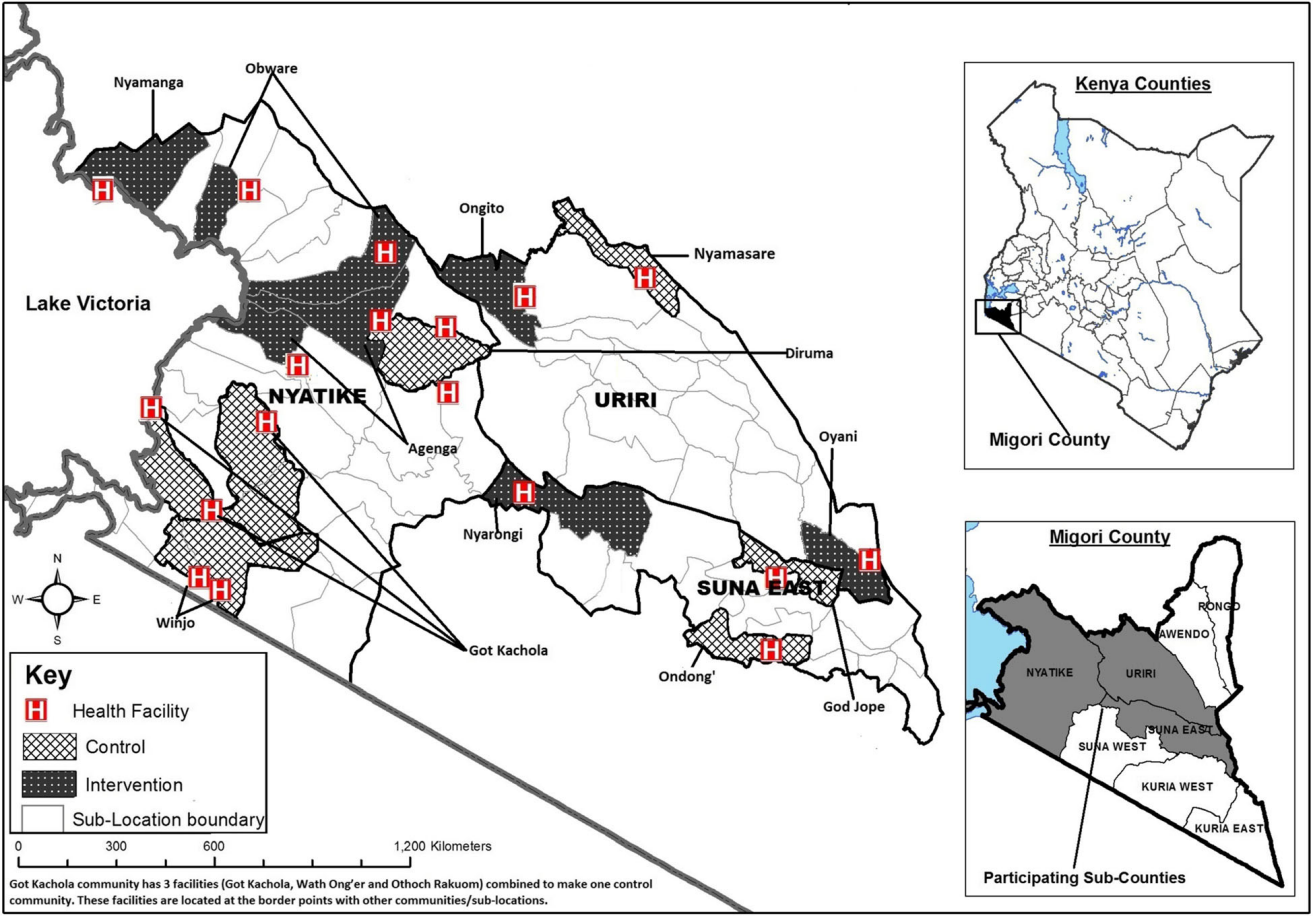
Map of communities randomized to control and intervention activities in Migori, County Kenya. This map was developed by Easter Olwanda, who has provided written permission for use in this publication

After community enumeration, training and protocol finalization, we launched the cluster-randomized trial in the six control and six intervention communities in Migori County. (Figure 2) Phase 1 of the trial, consisting of the activities listed below, took place over the course of 1 year.

- **Outreach and education:** In all communities, information about cervical cancer screening and the opportunity to learn more about HPV-based testing were provided through community outreach, including fliers, posters and brief informational sessions in markets, churches and women’s group meetings. Women and community leaders were provided with information on how to access cervical cancer screening in their community, e.g. location of clinic or timing and location of community health campaign. In all communities, women were invited to participate in a brief, standardized cervical cancer education module, either at the health campaign or in the health facility. The module is approximately 15 min and covers topics ranging from simple anatomy, definition of cervical cancer and HPV, how screening works, what treatment is available for precancerous lesions, and how to perform HPV self-testing.
- **Community-based testing (intervention group):** In six communities randomized to community-based HPV testing, community outreach teams carried out two-week community health campaigns in which HPV-testing was offered through self-collection. In order to reach the entire community, the campaign moved to multiple sites over the two-week period, with approximately one to 2 days at each site. The campaigns consisted of health education and informed consent, after which a health worker provided additional instructions about self-collection and recorded a mobile phone number before dispensing the HPV testing kit. The woman then would go to a private area in the campaign tent to self-collect the specimen and returned the completed collection kit to the health worker prior to leaving.
- **Clinic-based testing (comparison group):** In the six communities randomized to the control arm (clinic-based testing), women were directed through community outreach to go to their local health facility during regular clinic hours to carry out screening. At the clinic, a health worker offered the educational module, obtained informed consent and provided additional information about self-collection, and recorded a mobile phone number before dispensing the HPV testing kit and instructions.
- **HPV testing:** While HPV test results are not the primary outcome for this study, the accuracy and reproducibility of measurements are essential for outcomes in both arms and for modeling the impact of the implementation strategies in larger populations. We tested the DNA for 14 HPV types (16, 18, 32, 33, 35, 39, 45, 51, 52, 56, 58, 59 and 68) using CareHPV™ testing system. Collected specimens were transported daily from the CHCs and weekly from the health facilities to the study lab at Migori County Hospital. Tests were run in batches of 90, with a turnover time of approximately 1–2 weeks for results.
- **Notification of HPV results:** HPV test results were preferentially sent to women via text message with instructions about appropriate follow-up as recommended by the Kenya Ministry of Health guidelines. Messages were developed by key stakeholders and women from the target population during the focus group discussions. Women who did not have access to a phone, or did not wish to receive their results by SMS could opt for a return visit to the clinic, or a home visit.
- **Standard referral for treatment (both arms, Phase 1):** Women who were HPV-positive were referred to Migori County Hospital for a visual exam with acetic acid and treatment with cryotherapy per the WHO guidelines [44].
- **In-depth participant interviews and focus groups:** We conducted semi-structured interviews with randomly selected participants in both arms at three key points in the cervical cancer prevention cascade: screening delivery (*n* = 30), notification of results (n = 30) and treatment access (all). Participants were contacted either in person or by phone, and interviews conducted in person by experienced qualitative interviewers in the local language using interview guides developed by the research team. Topics explored in these interviews will elucidate ways to make cervical cancer screening more acceptable and accessible to women. Interviews captured quantitative data about women’s participation in various aspects of the prevention cascade. Interviewers then explored women’s perspectives of their experience with the intervention and explanatory factors related to the decisions to access screening and treatment through open-ended questions. Among women who did not access treatment, we probed for factors or strategies that would allow them to link to care in the future.
- **Provider and key stakeholder interviews and focus groups:** During the cluster- randomized trial, providers in the community health campaigns underwent brief interviews at two time points: after three and six campaigns had been completed. In the clinic-arm, providers underwent interviews at three, 6 and 12 months into the intervention. These interviews will help to understand explanatory factors for the success or failure of the intervention from a health system perspective. Interview topics included personal attitudes and beliefs around screening importance and feasibility, perceived and actual barriers to implementation and potential strategies to overcome provider, health delivery and patient-level barriers to screening and treatment.

#### Intervention development and cluster randomized trial: Phase 2

- **Enhanced linkage to treatment:** We will work with health care providers, community members and other stakeholders to review outcomes from the quantitative, qualitative and process measures in Phase 1, critically examine and modify the cervical cancer *screening* strategy to develop and pilot the enhanced linkage to *treatment* intervention over a 6–9 month period between Phase 1 and 2. We will do this through a series of key stakeholder meetings, followed by the establishment of smaller working groups for the creation of the specific intervention components. In the first set of meetings, we will present the findings from Phase 1 and seek feedback on representativeness and discuss implications for culturally relevant intervention strategies. Options for the most feasible and acceptable strategies to increase the number of women linking to treatment will be explored in the light of that data.

Criteria for potential strategies include: community-developed, low-cost, feasible in all study communities, and able to ensure treatment for HPV-positive women in a timely manner in accordance with Ministry of Health guidelines. In a second, small stakeholder meeting, we will review potential strategies discussed and developed in the first meeting, and discuss solutions proposed in other settings, including the use of mobile treatment units, transportation vouchers, and treatment “navigators” to help women understand and travel to treatment sites. Once the linkage intervention has been defined, we will hold another working group with stakeholders to create a standardized protocol, training manual, standard operating procedures and data collection instruments. After equipment procurement, provider training and further outreach messaging, the enhanced linkage strategy will be piloted in two to three Phase 1 intervention communities prior to launch of Phase 2.

#### Implementation framework

The study design, and outcome measures are centered in the essential implementation metrics as defined by the RE-AIM framework, which modified to the context of our study (Table 1) [17]. Outcomes will be evaluated through quantitative, qualitative and process measures. This design will allow us to test the following hypotheses:

- Community-based cervical cancer screening will reach a larger portion of eligible women and be more acceptable to patients and providers than clinic-based testing. (REACH)
- A community-driven intervention will improve linkage to treatment among women who need treatment after an HPV-positive screening test compared to standard referral. (EFFICACY)
- We will identify modifiable patient and health system challenges that can be addressed to make health campaign based HPV testing and enhanced linkage to treatment succeed and be sustainable. (ADOPTION & MAINTENANCE)
- Community-based cervical cancer screening with enhanced linkage to treatment will have a greater population-level health impact as measured in women reached with screening and any necessary treatment, and favorable cost-effectiveness profile compared to clinic-based strategies and standard referral for treatment. (IMPLEMENTATION)

**Table 1:** A modified RE-AIM framework to evaluate community health campaign-based cervical cancer screening compared to health-facility based screening

	Dimension Goal	Implementation Question	Hypothesis
Reach	Who is intended to benefit?	How do we reach reproductive-aged women in rural kenya?	A screening strategy offered through community health campaigns in a central location will reach a large proportion of reproductive-aged women.
	How do we reach them?		
Effectiveness	Is the program effective?	Are women getting screened for cervical cancer with HPV?	A community-based strategy allowing for self-testing will be highly acceptable.
	How do we ensure effectiveness?	Are HPV + women successfully linking to treatment?	Innovative, patient and provider-designed strategies will increase the number of women linking to care.
Adoption and Maintenance	How can strategy be maintained after initial implementation and adopted in similar communities?	What are the patient, provider and delivery system processes necessary to ensure consistent service provision?	A screening protocol with a simple, patient-performed test offered as part of a health fair will minimize the costs to the health care system to introduce screening.
	What are the short and long-term health effects in the community?	What is the population-level health impact of screening using HPV self-testing in the CHCs with enhanced linkage to care?	The high number of at -risk women reached through the CHC-base strategy with enhanced linkage to care would produce a greater population-level health impact.
Implementation	What is adherence to the implementation strategy at the delivery level?	Is HPV testing being offered and delivered consistently at the CHC and clinic sites?	Providing testing in a high-volume CHC will reach a large number of women with low staffing and infrastructure needs, and will therefore have a lower cost per woman treated than a standard strategy.
	What are the costs of implementation?	What is the cost per lesion treated?	

#### Participants

Our target population is women living in rural Kenya who are eligible for and would benefit from cervical cancer screening per the Kenya Ministry of Health Guidelines (25–65 years old with an intact uterus and cervix). The study population is women 25–65 years in the twelve communities in the Nyanza Province who access screening during both phases of the trial.

#### Recruitment and consent

Communities participating in the trial provided verbal assent in the planning process with written consent obtained from individuals for screening. Participants were recruited through the community health campaigns and in the clinics. Women within the target age range were invited to attend the cervical cancer educational module. After the health talk, women were asked to provide informed consent by research assistants for a post-module questionnaire and follow-up after screening completion. Women who were not willing to provide informed consent were still able to attend the health talk and have access to the HPV screening strategy assigned to her arm, but were not contacted for the follow-up in-depth interviews or participation in focus group discussions.

#### Primary and secondary endpoints

To determine the **reach** of cervical cancer screening using HPV-testing in community health campaigns compared to clinics, we are using the following metrics: i) the absolute number of women who completed screening in each arm and ii) the proportion of women in each arm who completed screening. The total number of women in each arm is the number of women 25–65 in each community as determined by census data. Secondary outcomes will include iii) the proportion of women who accept screening among women offered at each site and iv) the proportion of women in the clinic-based arm who request clinician-collected specimens.

To determine the **efficacy** of a community-developed strategy to increase treatment access, we will compare the efficacy of the community-based HPV testing with standard versus enhanced linkage to treatment using the following metrics: i) the number of women who receive treatment after screening HPV + in the intervention (Phase 1) compared to the enhanced intervention (Phase 2) and ii) the proportion of HPV-positive women in each arm who complete treatment. Secondary outcomes that address quality of care concerns for the models will include iii) the proportion of women who receive the correct treatment (per Ministry of Health protocol) during a single treatment visit and iv) the average time between HPV testing and access of treatment by arm.

#### Data collection

We collected data on both screening and linkage to treatment from both control and intervention communities in Phase 1. Data was collected by members of the research team and entered into pre-programmed tablet computers using OpenDataKit software (ODK™),(https://opendatakit.org) which had been used by this research team for the past several years. Programming included checks for range, structure, and internal consistency. During the community-health campaigns, data was collected directly from providers and participants into the tablets and was transferred daily via a secure electronic transfer to our data center facility in Kisumu, Kenya and stored on a secure server. To capture visits and outcomes from clinic-based screening and facility treatment, a member of the research team visited each clinical facility on a weekly basis to enter data from Ministry of Health registers and study-specific forms into the tablets. Data transfer from clinical-sites took place weekly. The same data collection procedures will be applied in Phase 2.

#### Implementation consistency

All four basic components of the intervention (outreach/education, HPV-testing, notification of results and linkage to treatment) were monitored throughout Phase I of the trial to ensure maintained fidelity to the protocol and quality of service and message delivery. Quantitative outcome measures, as well as the process measures listed below assessed continued fidelity of the intervention as offered. Qualitative data from in-depth interviews, focus-group discussions and process measures will provide a more complete picture about subtle but important factors that may influence the actual service delivery and uptake.

#### Sample size

Using estimates from the 2009 Kenya census [45] with projected population growth for 2015 and results from recent community-wide census enumeration carried out by a cluster randomized trial in an adjacent district [46], we have estimated the total available population of women to be approximately 1000 per community of 5000. The estimates for attendance at community health campaigns (60% or 600 women) and clinics (30% or 300 women) and screening uptake were based on our formative work, community health campaign attendance in adjacent districts, and prior studies of self-collected HPV testing [27, 28, 47]. Our assumptions were that (1) attendance would be higher at community health campaigns and (2) screening uptake would be higher among women attending community health campaigns because most women attend based on outreach messaging around cervical cancer. These assumptions suggested a study population of 510 women per community accessing testing through community health campaigns and 210 accessing screening through clinics. For Phase 1, the total number of women accessing screening in the six communities randomized to community health campaigns would be 3060 and 1260 in communities randomized to clinic-based testing. We used these conservative estimates for sample size calculations (see below), but allocated resources for up to 4500 women in the community-testing arm and 2000 women in the clinic-based screening arm to ensure continuity of study activities. We also enrolled all providers and targeted key ministry of health stakeholders for quantitative and qualitative assessments of barriers and facilitators to care. A representative subset of this group were invited to participate in meetings to develop the enhanced linkage intervention.

#### Statistical analysis

- *Preliminary Analysis:* For each outcome, we will produce descriptive statistics (frequencies, proportions, etc.) overall, across clusters of interest (community, clinic, provider, etc.), and over time. We will also graph these data to identify visual trends.
- *Primary and Secondary Analysis:* Although this is a community-level intervention, the main outcomes will be analyzed at the individual level. We will compare the number and proportion of women who screen for HPV (reach) and who get treated for a positive HPV test (efficacy) in communities assigned to community vs. clinic-based testing using generalized estimating equations to account for the correlation among observations within communities. Efficacy: We will employ a log link and Poisson distribution with an offset term to represent the size of each community.
- *Power calculations:* We anticipate being able to observe a 30% difference in overall screening uptake between the control and intervention arms, a conservative estimate relative to previous cluster randomized trials. The power calculations assume an alpha of 0.05, a beta of 0.20, and an intracluster correlation coefficient of 0.072 for screening and 0.11 for treatment, based on calculations from a cluster-randomized trial of HPV efficacy [5].

#### Cost-effectiveness analysis (maintenance)

We assessed the costs, population health impact, and incremental cost effectiveness of three intervention strategies (clinic-based screening with standard linkage to treatment; community screening with standard linkage; and will assess community screening with enhanced linkage). To do this, we undertook a micro-costing of the resources needed to carry out the activities in both arms in Phase 1 and 2. Costing included 1) personnel (including fringe benefits); 2) recurring supplies and services; 3) capital and equipment; and 4) facility space. Intervention costs were assessed using a uniform cost data collection protocol to quantify resources used and associated costs in each of the study sites (community-health campaigns, clinics, laboratories and district hospitals). Data was obtained through administrative record review and interviews with administrative, finance and human resources staff, supplemented by direct observation in a limited number of staff “time and motion” studies in order to distinguish cervical cancer-related activities from other health services delivered by the same personnel. Costs were summarized as total program costs as well as costs per woman screened and per HPV positive women treated.

We observed study outcomes to estimate the health outcomes associated with each screening and linkage strategy. Observed data include will include the number of women screened and treated for high-risk HPV, the proportion of women undergoing cryotherapy vs. LEEP, and the side effects associated with each treatment. We will find the best possible available data to estimate the prevalence of various HPV-subtypes in the region, and the associated risks of cervical intraepithelial neoplasia (CIN), recurrence rates of CIN after treatment, and invasive cancer in women with and without treatment. We will translate each health event into a standard metric of burden of disease, Disability-Adjusted Life Years (DALYs), which combines morbidity (and associated disability) with premature mortality (lost “life years”).

We will use the micro-costing described above to estimate several indices comparing costs to desired program outcomes: cost per case of HPV detected; cost per case of CIN detected; cost per woman successfully linked to facilities for treatment; and cost per woman treated. We will construct a decision model to estimate the health impact of HPV screening and linkage to treatment in a population cohort of 1000 women. This model will explicitly portray the paths from HPV to detection (by clinical presentation or screening), the risks of clinical progression, and outcomes with and without treatment (early or late). It will incorporate data on local epidemiology (HPV prevalence and cervical cancer, from Phase 1 and existing surveillance data); the clinical course of HPV and cervical cancer (from scientific literature); and the effectiveness of treatment (with cryotherapy and LEEP, as well as for more advanced disease, from scientific literature). Model outcomes will include deaths from cervical cancer, lost years of life, and morbidity (short and long-term), and DALYs (disability adjusted life years).

We will use the decision-analysis model to assess the incremental cost-effectiveness ratio (ICER), defined as the added cost per DALY averted, when comparing intervention strategies. We will also calculate the ICER compared to the current standard, which is no organized available screening, using baseline data of screening availability and use to calculate this. We will also estimate the costs for scaled-up replication, which will include variations in the number of and costs for personnel, community-health campaign structure and duration, HPV screening test costs laboratory costs and different linkage strategies.

#### Process measures

**Fig. 3:**
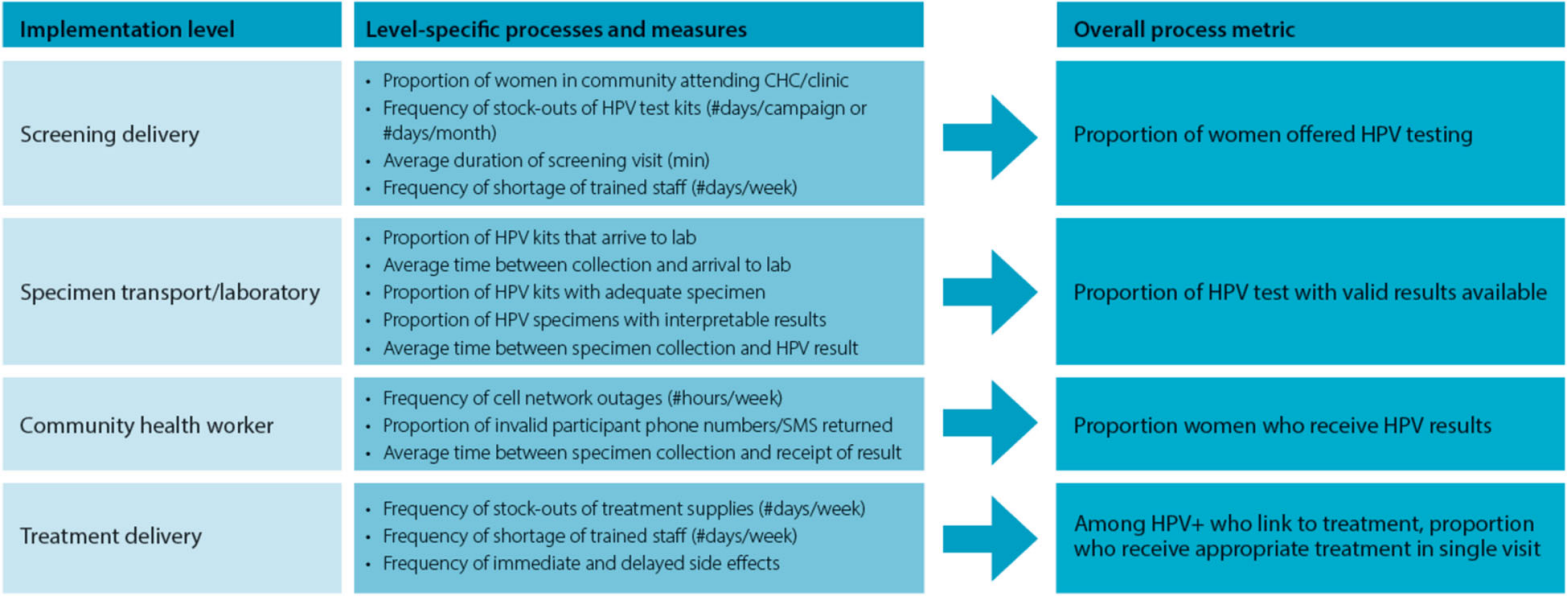
Quantitative process measures for four aspects of cervical cancer prevention program delivery

We will use quantitative process analyses to evaluate the strategy implemented in both arms at four levels of the intervention delivery process (Fig. 3). These include a) the proportion of women from each community health campaign offered HPV testing or referral (community health campaign-level processes); b) the proportion of HPV tests for which valid results are available (specimen transport and laboratory processes); c) the proportion of women who receive their test results (community health worker processes); and c) the proportion of HPV+ women attending a treatment visit who receive the appropriate treatment per Kenya Ministry of Health guidelines (health delivery center processes). We will use data from the provider and participant interviews and focus groups to explore the factors impacting the relevant service delivery processes that affect the overall result though quantitative and qualitative measures as well as explore additional key barriers and facilitators to both screening and treatment. Focus group data will enrich these conclusions and be used to develop an enhanced linkage intervention.

### Ethical review

The trial was reviewed by an implementation and dissemination science section at the National Cancer Institute prior to funding. Ethical approval was obtained by the Committee for Human Research at the University of California, San Francisco (#14–13,698), Duke University Institutional Review Board (Pro0007742), and the Scientific and Ethical Review Unit at the Kenya Medical Research Institute (SERU 2918). Any major protocol changes will be communicated to all three review boards and to the trial registry at ClinicalTrials.gov. Complete trial registry data is available in a Additional file 1.

### Trial status

Focus Group Discussions and in-depth interviews with key informants took place in August and September 2015. A pilot campaign took place in December 2015. Screening activities and enrollment in Phase I of the cluster-randomized trial were carried out between January and September 2016. We are now sharing feedback of Phase I results and observations with various stakeholders (community members, health care providers, and health management teams) in preparation for FGDs and working groups, which are aimed at enabling us design a strategy for enhanced linkage to treatment.

## Discussion

Substantial progress toward cervical cancer prevention has been made through research validating low-cost screening strategies that have been included in national and international protocols and guidelines. However, like many international guidelines, the WHO cervical cancer guidelines lacks advice on active implementation strategies [48]. While this is partly due to an emphasis on the clinical portion of the guidelines, some of this can be attributed to the lack of effective implementation strategies. Our goal with this novel study is to work with the community using a rigorous implementation framework to develop a strategy that could be scaled to improve the reach and efficacy of cervical cancer prevention programs in rural Africa, where the lack of health care infrastructure and services has lead to poor health outcomes. We are also hoping that the methodology of this project can be expanded to develop implementation strategies that would help address other health care needs.

Based on our formative work, we expect that community-based cervical cancer screening will reach a substantially larger portion of eligible women than clinic-based testing. Our findings will help guide implementation and optimization of a community-based HPV testing model. While we anticipate that the community-driven enhanced intervention will be more effective at linking women to facilities for treatment than the standard referral system, this study will allow us to test both models and look at various aspects of implementation, including cost-effectiveness. In addition to these findings, we will provide a model for a successful strategy to link women to treatment within cervical cancer screening program and to provide program leaders and policymakers with a tool kit to design and evaluate a context-specific enhanced linkage strategy that could be implemented in their own settings. We expect that community-based HPV screening will have a greater cost effectiveness and public health impact than clinic-based testing, and that enhanced linkage strategies will amplify these differences. Overall, our findings will provide evidence to inform clinical protocols and government policy regarding the provision of cervical cancer prevention strategies and provide a guide for adaptation and evaluation of similar programs in other settings. Ultimately programs that both use evidence-based techniques and reach a large proportion of the population will impact the millions of women at risk for cervical cancer in low resource countries worldwide.

## Conclusions

This project will have broad implications at both local and national policy and planning levels, given the enthusiasm of the Kenya Ministry of Health and Division of Reproductive Health to implement national cervical cancer prevention strategies and their partnership in this project. When the analyses are complete, we will have produced a comprehensive description of barriers and facilitators to providing clinic and community-based cervical cancer screening through HPV testing, determined which strategy has greater reach and a better cost-effectiveness profile, and developed a strategy to improve linkage to treatment in partnership with the community. If a community-based screening strategy is shown to have more reach with a favorable cost-effectiveness profile, this could be a viable strategy for roll-out in similar settings in Kenya and possibly for adaptation to other East African countries with a high cervical cancer burdens. Just as importantly, if community-based testing is more effective and scalable than clinic-based testing, we will explore factors necessary to improve access to clinic for cervical cancer screening and other preventive care services.

## Additional file

Additional file 1: WHO Trial Registry Data. (PDF 86 kb)

## Abbreviations

CHC: Community health campaign
CIN: Cervical intraepithelial neoplasia
DALY: Disability adjusted life year
FGD: Focus group discussion
HPV: Human papillomavirus
ICER: Incremental cost-effectiveness ratio
LEEP: Loop electrosurgical excision procedure
RE-AIM: Reach, effectiveness, adoption, implementation and maintenance (Implementation Framework)
WHO: World health organization
