# Supplementary material for: Study protocol for a cluster-randomized trial to compare human papillomavirus based cervical cancer screening in community-health campaigns versus health facilities in western Kenya
Source: BMC Cancer. 2017 Dec 6;17:826. doi: 10.1186/s12885-017-3818-z (PMC5717798; doi:10.1186/s12885-017-3818-z)
Supplement: Additional file 1: — WHO Trial Registry Data. (PDF 86 kb) [file 12885_2017_3818_MOESM1_ESM.pdf]

WHO Trial Registry Data

Data Category	Information
Primary registry and trial identifying number	ClinicalTrials.gov, NCT02124252
Date of registration in primary registry	25 April 2014
Secondary identifying numbers	R01CA188248
Source of monetary of material support	National Institutes of Health/National Cancer Institute
Primary sponsor	National Institutes of Health/National Cancer Institute
Secondary sponsor	n/a
Contact for public or scientific queries	Megan Huchko, MD, MPH, megan.huchko@duke.edu
Public Title	Evaluating a community-driven cervical cancer prevention protocol in western Kenya
Scientific Title	Evaluating a community-driven cervical cancer prevention protocol in western Kenya
Country of recruitment	Kenya
Health Condition Studied	Cervical cancer/HPV infection
Intervention	Human papillomavirus-based cervical cancer screening offered in community health campaigns
Key Inclusion and exclusion criteria	Women, aged 25-65 living in target communities in western Kenya
Study type	Cluster randomized trial
Date of first enrollment	January 2016
Target sample size	7500
Recruitment status	Recruiting
Primary outcomes	Reach and efficacy of community-health campaign based screening
Key secondary outcomes	Cost and cost-effectiveness of the two implementation strategies; process measures associated with more effective service delivery
